# Excess Child Mortality Associated With Colombia’s Armed Conflict, 1998-2019

**DOI:** 10.1001/jamanetworkopen.2024.8510

**Published:** 2024-04-26

**Authors:** Caitlin A. Moe, Andrés Villaveces, Pablo Montoya, Ali Rowhani-Rahbar

**Affiliations:** 1 Department of Epidemiology, University of Washington, Seattle; 2 Department of Global Health, University of Washington, Seattle; 3 Department of Pulmonary and Critical Care Medicine, University of California, Irvine; 4 Department of Pulmonary and Critical Care Medicine, University of California, San Francisco; 5 National Center for Injury Prevention and Control, Centers for Disease Control and Prevention, Atlanta, Georgia; 6 Sinergias Alianzas Estratégicas para la Salud y el Desarrollo Social, Bogotá, Colombia; 7 Firerarm Injury Prevention and Research Center, University of Washington, Seattle

## Abstract

**Importance:**

Armed conflicts are directly and indirectly associated with morbidity and mortality due to destruction of health infrastructure and diversion of resources, forced displacement, environmental damage, and erosion of social and economic security. Colombia’s conflict began in the 1940s and has been uniquely long-lasting and geographically dynamic.

**Objective:**

To estimate the proportion of infant and child mortality associated with armed conflict exposure from 1998 to 2019 in Colombia.

**Design, Setting, and Participants:**

This ecological cohort study includes data from all 1122 municipalities in Colombia from 1998 to 2019. Statistical analysis was conducted from February 2022 to June 2023.

**Exposure:**

Armed conflict exposure was measured dichotomously by the occurrence of conflict-related events in each municipality-year, enumerated and reported by the Colombian National Center for Historic Memory.

**Main Outcomes and Measures:**

Deaths among children younger than 5 years and deaths among infants younger than 1 year, offset by the number of births in that municipality-year, enumerated by Colombia’s national vital statistics.

**Results:**

The analytical sample included 24 157 municipality-years and 223 101 conflict events covering the period from 1998 to 2019. Overall, the presence of armed conflict in a municipality was associated with a 52% increased risk of death for children younger than 5 years of age (relative risk, 1.52 [95% CI, 1.34-1.72]), with similar results for 1- and 5-year lagged analyses. Armed conflict was associated with a 61% increased risk in infant (aged <1 year) death (relative risk, 1.61 [95% CI, 1.43-1.82]). On the absolute scale, this translates to a risk difference of 3.7 excess child deaths per 1000 births (95% CI, 2.7-4.7 per 1000 births) and 3.0 excess infant deaths per 1000 births (95% CI, 2.3-3.6 per 1000 births) per year, beyond what would be expected in the absence of armed conflict. Across the 22-year study period, the population attributable risk was 31.7% (95% CI, 23.5%-39.1%) for child deaths and 35.3% (95% CI, 27.8%-42.0%) for infant deaths.

**Conclusions and Relevance:**

This ecological cohort study of Colombia’s spatiotemporally dynamic armed conflict suggests that municipal exposure to armed conflict was associated with excess child and infant deaths. With a record number of children living near active conflict zones in 2020, policy makers and health professionals should understand the magnitude of and manner in which armed conflicts directly and indirectly undermine child health.

## Key Points

**Question:**

What is the association between armed conflict and child mortality in Colombia?

**Findings:**

In this ecological cohort study of all municipalities in Colombia from 1998 to 2019, the presence of armed conflict was associated with a 52% increased risk of child (aged <5 years) mortality and a 61% increased risk of infant (aged <1 year) mortality. Over the 22-year study period, 32% of child deaths and 35% of infant deaths in Colombia could be associated with armed conflict.

**Meaning:**

This study suggests that armed conflict–related events are directly and indirectly associated with an increase in child mortality rates in Colombia.

## Teaser

This ecological cohort study estimates the proportion of infant and child mortality associated with armed conflict exposure from 1998 to 2019 in Colombia.

## Introduction

Worldwide, an estimated 452 million children—more than 1 in 6—live within 50 km of active war zones.^1^ Armed conflict, defined as armed violence between organized groups, is a major cause of injury and death worldwide, posing a significant public health problem.^2,3^ Beyond mortality directly resulting from violence, armed conflicts indirectly harm health through more subtle means. The destruction of health infrastructure, diversion of resources, forced displacement, environmental damage to water or sanitation access, and erosion of social and economic security introduce and exacerbate risk factors for more death and morbidity off the battlefield, particularly when civilian access to water, food, shelter, or health care are curtailed.^4,5^ Young children, while rarely combatants, are some of the most vulnerable to both direct and indirect effects of armed conflict.^6^

Colombia has experienced over 7 decades of armed conflict, beginning in the 1940s and evolving over time to include multiple state, nonstate, and pseudostate (paramilitary) armed groups. The conflict intensified in the 1990s due to the increase in drug trafficking and mobilization of paramilitaries in the 1980s.^7,8^ Of the 9.5 million Colombians registered as survivors of the conflict, 1.9 million (20%) were children (aged <18 years).^9^ Young children (aged <5 years) comprise 2% of those who experienced conflict-related trauma overall, and since 1985, nearly 200 000 children younger than 5 years were killed, abducted, disappeared, or tortured.^9,10^ Despite the long-lasting armed conflict, Colombia has made substantial progress reducing mortality rates for children younger than 5 years from 36 per 1000 live births in 1990 to 13 per 1000 live births in 2021.^11^ However, the national rate masks significant subnational disparities^12^; the mortality rate for children younger than 5 years in rural areas is almost double the national average.^13^

Armed conflict also disrupts the collection of data and the ability to conduct public health programs and outreach; so, empirical estimates of the association of specific conflicts with child health are scarce. A limited body of research has examined the association of armed conflict with child health indicators, mostly in sub-Saharan Africa.^14,15,16,17,18,19,20^ However, the conflicts in these settings were generally short term (<5 years) and limited to specific areas.^21,22^ Reviews of the literature show that the empirical association of armed conflict with child health remains largely unknown, particularly in Latin America.^2,23,24^ By leveraging the spatial and temporal heterogeneity of Colombia’s armed conflict, this study estimated the association between armed conflict and infant and child survival over a period of 22 years.

## Methods

### Study Setting and Population

The unit of analysis for this ecological cohort study was municipality-year. Colombian municipalities are analogous to US counties; departments are analogous to US states. Colombia has a total of 1122 municipalities organized into 32 departments. Bogotá, the capital of Colombia, comprises its own municipality as a capital district. All municipalities were included in this study, although some municipalities (<1% overall) were excluded for some years because they did not exist yet or because the number of births was 1 or fewer. This study did not meet the University of Washington Human Subjects Division’s definition of human participants research because no data were collected from living individuals, all data were deidentified and publicly available, and no identifiable information was generated or collected; therefore, the requirement for informed consent was waived. This study followed the Reporting of Studies Conducted Using Observational Routinely-Collected Data (RECORD) statement.

The analytic sample consisted of 24 157 municipality-years during the period from 1998 to 2019. Municipality-years were included in the analysis if there were any deaths (among all ages) recorded to help ensure that death registry data were collected. The beginning of the study period was limited to 1998 because the current vital statistics program was implemented in 1997 and reached national coverage by 1998.^25^

### Measures

The main outcome measures, deaths among children under 5 years of age and deaths among infants younger than 1 year of age, were obtained from Colombia’s vital statistics microdata, which are collected, processed, and hosted by the National Administrative Department of Statistics (DANE in Spanish).^26^ DANE death records include data on age (by month for children <5 years), sex, municipality of residence, location of death, cause of death (recorded with *International Statistical Classification of Diseases and Related Health Problems, Tenth Revision* [*ICD-10*] codes), type of death certification, and other demographics, such as marital status and educational level for adult decedents. For this analysis, child deaths were measured as counts for each municipality-year, irrespective of cause, and were assigned to the child’s municipality of residence. Subgroupings of child deaths by cause were assessed a priori following standard classification for causes of death among infants and young children.^27^

The main independent variable, armed conflict, was based on counts of conflict-related events in each municipality-year. A municipality-year was considered exposed to armed conflict if there were any conflict-related incidents occurring in that municipality within the calendar year. Conflict data were obtained from the Colombian government’s National Center for Historical Memory (CNMH in Spanish), which was mandated by law to compile, process, and distribute data on the armed conflict.^28^ Data from the CNMH were collected from many sources, including the National Victims’ Registry (RUV in Spanish), the Justice Department, interviews with survivors, death records, media reports, and academic research, among others. The RUV is a database of self-enrolled survivors of the Colombian conflict or their families, applicable for events occurring since 1985, who are classified under a typology of 13 types of events (eMethods in Supplement 1). Survivors entered into this registry are entitled to receive humanitarian aid and longer-term reparation benefits from the government.^9^ Incidents perpetrated by an armed group member and/or related to territorial disputes were considered conflict-related events, which were ascertained, de-duplicated, and categorized by the CNMH into one of the following categories: military actions, selective assassinations, attacks on towns, terrorist attacks, damage to civilian infrastructure or property, massacres, kidnapping, forced disappearance, recruitment of children, sexual violence, and antipersonnel mines or unexploded munitions. Events that occurred simultaneously (eg, kidnapping at the same time as a massacre) were assigned to the category in which the event was first reported to the CNMH. Conflict events did not necessarily result in fatality; however, even nonfatal incidents can be threatening and may inhibit access to medical care.^6^

Consistent with prior literature, we took a parsimonious approach to model adjustment to avoid including factors that may be on the causal pathway between conflict and child mortality.^21,29^ Each model in this analysis was adjusted with 3 indicators for the occurrence of severe natural disasters requiring national emergency aid: volcano eruption and/or earthquake, hurricane and/or flooding, and droughts. These disasters may increase the likelihood of both armed conflict and child mortality and are not downstream effects of armed conflict. Disaster data were obtained from Colombia’s National Unit for Disaster Risk Management (UNGRD in Spanish), which included any event that was declared a public emergency and received support from the UNGRD’s National Emergency Fund.^30^

### Statistical Analysis

Statistical analysis was conducted from February 2022 to June 2023. Negative binomial regression models were constructed for each count outcome (number of deaths among infants aged <5 years and number of deaths among children aged <1 year) offset by the number of births in that municipality-year. Zero-inflated negative binomial models were considered for the large number of zeroes in the response variable; however, the nonzero inflated negative binomial regression model was chosen for better model fit.

The regression model equation is provided in the eMethods in Supplement 1. For the main analysis, the outcomes of deaths of children younger than 5 years and infant deaths were modeled with (1) concurrent-year conflict events, (2) conflict events lagged to 1 year prior, and (3) conflict events lagged to 5 years prior. Subgroup analyses were also performed of child deaths by cause of death as assigned using *ICD-10* codes in official death records.

Risk differences were computed using average marginal effects postestimation from the models, and population attributable risk (PAR) was estimated using the punaf function in Stata, version 17 (StataCorp LLC).^31^ Excess mortality is the number of deaths beyond what would be expected in the absence of exposure to armed conflict. Analyses were conducted in Stata, version 17, and the maps were prepared using R, version 4.3 (R Project for Statistical Computing).

## Results

**Figure 1.**
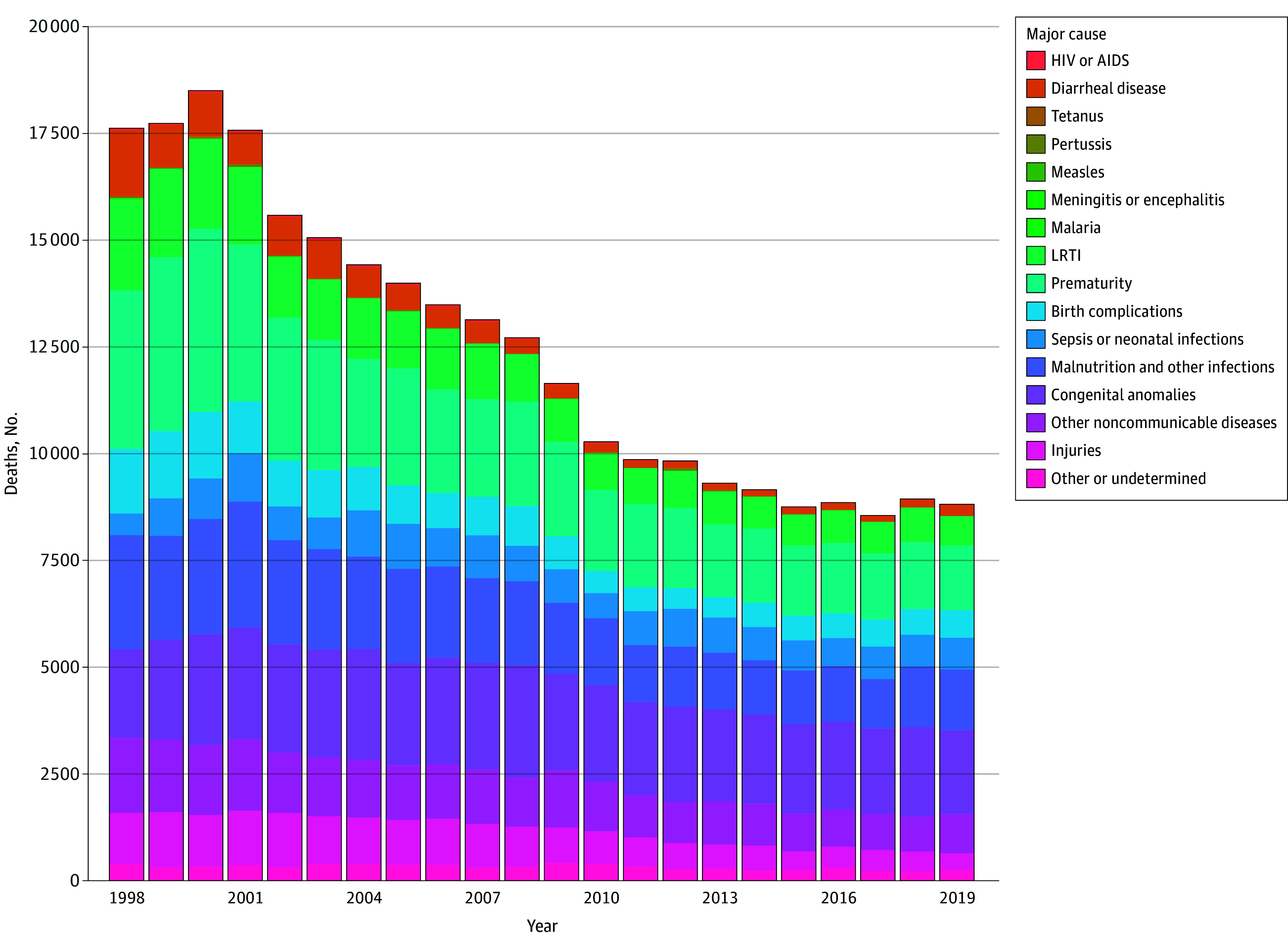
Child (Aged <5 Years) Deaths in Colombia by Cause, 1998-2019 LRTI indicates lower respiratory tract infection.

The analytic sample consisted of 24 157 municipality-years during the period from 1998 to 2019. A description of the analytical sample is shown in Table 1. The distribution of causes of child mortality over time are shown in Figure 1. The mean (SD) count of deaths of children younger than 5 years per municipality-year over the study period was 11 (86); 39.4% of municipality-years (9519 of 24 175) reported at least 1 death of any age but 0 deaths among children younger than 5 years of age. When parameterized as a child mortality rate per 1000 live births, the mean (SD) mortality rate for children younger than 5 years was 9.6 (18.3) per 1000 births. Among infants (aged <1 year), the mean (SD) count of deaths per municipality-year was 9 (74), and 49.5% of municipality-years (11 959 of 24 157) reported 0 infant deaths. The mean (SD) infant mortality rate was 6.5 (13.9) per 1000 births.

**Table 1.** Description of Analytical Sample in Colombia, 1998-2019

Characteristic	Conflict-affected municipality-years (n = 13 591)	Non–conflict-affected municipality-years (n = 10 566)	Colombian municipality-years overall (N = 24 157)
Total population, No.			
Mean (SD)	57 897 (312 964)	15 659 (32 939)	39 428 (236 705)
Median (IQR)	16 558 (9042-31 705)	8414 (4811-15 649)	12 362 (6628-24 769)
Births, No.			
Mean (SD)	951 (5036)	210 (500)	627 (3809)
Median (IQR)	247 (123-520)	102 (53-206)	167 (80-380)
Child (<5 y) deaths, No.			
Mean (SD)	19 (114)	2 (12)	11 (86)
Median (IQR)	2 (0-5)	0 (0-1)	1 (0-3)
Child (<5 y) mortality rate per 1000 births			
Mean (SD)	11.9 (16.6)	6.7 (19.9)	9.6 (18.3)
Median (IQR)	7.6 (0-16.0)	0 (0-8.2)	4.8 (0-13.0)
Infant (<1 y) deaths, No.			
Mean (SD)	16 (98)	1 (11)	9 (74)
Median (IQR)	1 (0-4)	0 (0-1)	1 (0-2)
Infant (<1 y) mortality rate per 1000 births			
Mean (SD)	8.3 (12.2)	4.3 (15.5)	6.5 (13.9)
Median (IQR)	4.6 (0-11.4)	0 (0-4.1)	1.1 (0-8.7)
Armed conflict events, No.			
Mean (SD)	16 (48)	0	9 (37)
Median (IQR)	5 (2-15)	0	1 (0-6)
Hurricane or flood, No. (%)	5847 (43.0)	3769 (35.7)	9616 (39.8)
Volcano or earthquake, No. (%)	2175 (16.0)	1990 (18.8)	4165 (17.2)
Drought, No. (%)	133 (1.0)	209 (2.0)	342 (1.4)

**Figure 2.**
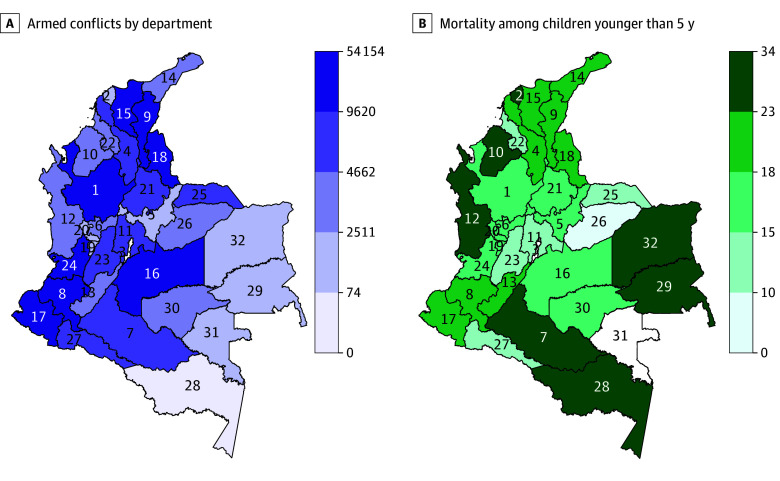
Armed Conflict Events and Child Mortality in Colombia, 1998-2019 A, Total number of armed conflict events by department. B, Mean department-level child (<5 years) mortality rate, 1998-2019. Department key: (1) Antioquia; (2) Atlántico; (3) Bogotá, D.C.; (4) Bolívar; (5) Boyacá; (6) Caldas; (7) Caquetá; (8) Cauca; (9) Cesar; (10) Córdoba; (11) Cundinamarca; (12) Chocó; (13) Huila; (14) La Guajira; (15) Magdalena; (16) Meta; (17) Nariño; (18) Norte de Santander; (19) Quindio; (20) Risaralda; (21) Santander; (22) Sucre; (23) Tolima; (24) Valle del Cauca; (25) Arauca; (26) Casanare; (27) Putumayo; (28) Amazonas; (29) Guainía; (30) Guaviare; (31) Vaupés; (32) Vichada. The islands representing the department San Andrés and Providencia are not shown but were included in analyses.

Although the data on conflict event counts were also heavily skewed, only 23 municipalities (2.0%) had 0 conflict-related events over the entire study period (10 municipalities in the Boyacá department, 6 in Guainía, 3 in Amazonas, 2 in Cundinamarca, and 2 in Santander; see Figure 2). The mean (SD) number of armed conflict events was 9 (37) per municipality-year, with a total of 223 101 conflict events from 1998 to 2019 in all municipalities. Of 24 157 municipality-years, 10 566 years (43.7%) had 0 conflict events.

Results from regression analyses are presented in Table 2. Overall, exposure to armed conflict was associated with a 52% increased risk (relative risk [RR], 1.52 [95% CI, 1.34-1.72]) for child (<5 years) deaths in affected municipalities in that year. Similarly, municipal exposure to armed conflict was associated with a 61% increased risk of infant (<1 year) deaths (RR, 1.61 [95% CI, 1.43-1.82]). Results were robust to exclusion of large cities (eTable in Supplement 1). On the absolute scale, we observed 3.7 excess child deaths per 1000 births (95% CI, 2.7-4.7 per 1000 births) and 3.0 excess infant deaths per 1000 births (95% CI, 2.3-3.6 per 1000 births) in that period, beyond what would be expected in the absence of armed conflict. Across the 22-year study period, the PAR was 31.7% (95% CI, 23.5%-39.1%) for child deaths and 35.3% (95% CI, 27.8%-42.0%) for infant deaths. In other words, over 30% of child deaths in Colombia from 1998 to 2019 were associated with the conflict; this translates to excess mortality of 86 743 (95% CI, 64 304-106 992) deaths among children younger than 5 years, including 79 351 (95% CI, 62 492-94 412) deaths among infants younger than 1 year.

**Table 2.** Associations of Conflict Exposure With Child and Infant Deaths, 1998-2019, Contemporaneous and Lag Periods^a^

Period	RR (95% CI)	RD (95% CI)^b^
Contemporaneous (same year)		
Child (<5 y) mortality	1.52 (1.34-1.72)	3.7 (2.7-4.7)
Infant (<1 y) mortality	1.61 (1.43-1.82)	3.0 (2.3-3.6)
1-y Lag		
Child (<5 y) mortality	1.51 (1.33-1.72)	3.5 (2.5-4.4)
Infant (<1 y) mortality	1.60 (1.40-1.81)	2.7 (2.1-3.4)
5-y Lag		
Child (<5 y) mortality	1.58 (1.39-1.81)	3.2 (2.4-4.0)
Infant (<1 y) mortality	1.69 (1.47-1.94)	2.5 (2.0-3.1)

Abbreviations: RR, relative risk; RD, risk difference.

^a^ All models adjusted for natural disaster indicators of hurricane or flood, volcano or earthquake, or drought and year fixed effects.

^b^ Computed per 1000 births.

When conflict exposure was lagged to correspond to conflict from 1 year prior, the RRs did not change. However, when lagged to 5 years prior, the RRs were slightly higher (Table 2). When examining the association of armed conflict with cause-specific child mortality, we observed some differences. As shown in Table 3, exposure to armed conflict was most strongly associated with deaths from malaria (RR, 5.54 [95% CI, 2.80-10.94]), diarrheal disease (RR, 2.13 [95% CI, 1.60-2.84]), and sepsis or newborn infections (RR, 2.22 [95% CI, 1.83-2.69]). The association with deaths due to malnutrition was also high (RR, 1.95 [95% CI, 1.76-2.16]), and the RR for deaths due to injury was not statistically significant at 0.96 (95% CI, 0.79-1.18). Among broader categories of underlying cause, we saw the largest associations between armed conflict and deaths due to communicable diseases, followed by noncommunicable diseases, for both children and infants.

**Table 3.** Child Mortality Rates by Cause of Death and Exposure to Armed Conflict in Colombia, 1998-2019^a^

Cause of death	Mean child mortality rate per 1000 live births (95% CI)		Relative risk (95% CI)		
	Conflict-affected municipality-years (n = 13 591 [56.3%])	Non–conflict-affected municipality-years (n = 10 566 [43.7%])	Contemporaneous	1-y Lag	5-y Lag
All cause	11.85 (11.79-11.91)	6.72 (6.67-6.77)	1.52 (1.34-1.72)	1.51 (1.33-1.72)	1.58 (1.39-1.81)
Diarrheal disease	0.87 (0.85-0.88)	0.31 (0.30-0.32)	2.13 (1.60-2.84)	2.01 (1.48-2.71)	2.33 (1.51-3.59)
Pertussis	0.007 (0.006-0.009)	0.004 (0.003-0.005)	2.14 (1.32-3.45)	4.52 (2.43-8.43)	16.80 (2.99-94.49)
Malaria	0.09 (0.08-0.09)	0.006 (0.005-0.008)	5.54 (2.80-10.94)	2.23 (0.75-6.66)	2.78 (0.40-19.08)
Lower respiratory tract infection	1.29 (1.27-1.31)	0.73 (0.72-0.75)	1.72 (1.52-1.94)	1.84 (1.64-2.07)	1.85 (1.58-2.17)
Prematurity	1.47 (1.45-1.49)	0.63 (0.62-0.65)	1.79 (1.55-2.07)	1.83 (1.47-2.14)	2.00 (1.67-2.38)
Intrapartum complications	1.13 (1.11-1.14)	0.78 (0.76-0.80)	1.47 (1.24-1.75)	1.41 (1.16-1.71)	1.55 (1.25-1.93)
Sepsis or newborn infections	0.37 (0.36-0.38)	0.12 (0.11-0.13)	2.22 (1.83-2.69)	2.25 (1.86-2.72)	2.46 (1.84-3.29)
Malnutrition and other infectious causes of death	1.93 (1.91-1.96)	0.85 (0.83-0.87)	1.95 (1.76-2.16)	1.86 (1.66-2.08)	2.02 (1.70-2.40)
Congenital anomalies	1.05 (1.04-1.07)	0.56 (0.55-0.58)	1.66 (1.41-1.96)	1.71 (1.44-2.03)	1.78 (1.47-2.14)
Other noncommunicable diseases	1.22 (1.21-1.24)	0.69 (0.67-0.70)	1.53 (1.33-1.76)	1.63 (1.40-1.88)	1.63 (1.36-1.95)
Injuries	1.91 (1.88-1.93)	1.65 (1.63-1.68)	0.96 (0.79-1.18)	0.98 (0.78-1.22)	1.00 (0.84-1.21)

^a^ All models were adjusted for natural disaster indicators of hurricane or flood, volcano or earthquake, or drought and year fixed effects.

## Discussion

In this ecological cohort study, we found a significant association of Colombia’s armed conflict with child and infant mortality rates. Overall, a municipality’s exposure to armed conflict was associated with a 52% increase in child mortality in that year. Nearly 1 in 3 child deaths during the study period was associated with the armed conflict; this translates to excess mortality of 86 743 (95% CI, 64 304-106 992) deaths among children younger than 5 years, including 79 351 (95% CI, 62 492-94 412) deaths among infants younger than 1 year between 1998 and 2019—fatalities that could have been prevented in the absence of armed conflict.

It is well documented that child and infant mortality rates are higher in countries or regions experiencing conflict.^23^ However, the association is confounded by countries in conflict more often experiencing other risk factors for child mortality, including lower gross domestic product, poor state infrastructure, and general socioeconomic development indicators.^32^ Upstream determinants of armed conflict include socioeconomic inequality and disenfranchisement but also arms proliferation and availability.^33^

Research on the population health effects of armed conflicts have focused on shorter-duration conflicts, such as those in Syria and Iraq, and multicountry studies have focused on conflicts in sub-Saharan Africa.^23,24^ An analysis of armed conflicts in 35 African countries between 1995 and 2015 found that violent clashes within 50 km during a child’s first year of life were associated with higher risk of infant mortality (<1 year) than in the absence of conflict.^23^ Long-term conflicts—those that lasted at least 5 years—were associated with the highest increase in infant mortality.^23^

There is less agreement on how long the effects of armed conflict last. In the multicountry analysis, infant mortality risk was elevated for up to 8 years after the conflict event.^23^ In our lagged analysis, we found largely consistent associations between armed conflict incidents and child and infant mortality. This difference in findings may be due to some unique characteristics of Colombia’s armed conflict, such as widespread exposure, long-term persistence, and massive population displacement. In 2018, 40% of Colombians reported being survivors of the armed conflict, defined as having been forcibly displaced or having a family member killed or kidnapped due to the conflict.^34^ It may be more difficult to disentangle the effects of repeated or widespread exposures to the long-term armed conflict in Colombia compared with those in sub-Saharan Africa.

Studies have examined the association of Colombia’s conflict with demographic indicators, including fertility,^35^ contraception use,^36^ educational outcomes,^37^ and maternal and infant health indicators.^29^ In their mixed-methods study, Ramos Jaraba et al^29^ did not find statistically significant differences in infant mortality between conflict-affected and non–conflict-affected municipalities but did observe increased rates of maternal mortality in municipalities with higher levels of conflict. In support of these findings, we found that deaths due to neonatal period–related causes, such as prematurity, intrapartum complications, sepsis, and other newborn infections, were some of the causes most strongly associated with conflict. The delivery of health services to pregnant women is necessary for both maternal and infant health outcomes but is crucial in conflict-affected areas. The availability of emergency obstetric services in particular was found to be critically affected by armed conflict in Colombia and Mexico.^29,38^

We also observed associations between exposure to armed conflict and child deaths due to malnutrition and diarrheal disease. These findings align with previous knowledge about the effects of armed conflict on children; food insecurity, curtailed access to clean water, poor sanitation, and displacement make children highly vulnerable to acute and infectious causes of death.^39^

### Limitations

These findings should be taken in view of some limitations. First, the exposure to armed conflict is likely underestimated. Although the CNMH, the RUV, and, more recently, the Comisión de la Verdad in Colombia have made efforts to identify, de-duplicate, and categorize conflict data, an inherent aspect of armed conflicts is that many events will go unreported in cases in which, for example, there are no survivors. Another aspect of intense violence is the difficulty in disentangling discrete incidents from each other. Our observed results are likely attenuated due to underestimated conflict events; in other words, the true association between exposure to armed conflict in Colombia and child mortality is likely higher than we observed. In addition, we included natural disasters as covariates in our analyses but did not include other confounders, such as socioeconomic indicators, so there may be some residual confounding. In our conceptual framework, poverty mediates the association between armed conflict and child mortality at the municipality level, and we wanted to include direct and indirect (ie, mediated) pathways for this association.

We also relied on official death registries for child mortality data. Although Colombia’s vital statistics have been validated, they may be less reliable (eg, missing births or deaths and/or less accurate assignation of causes of death), particularly in areas not controlled by the state during the armed conflict. To help address this limitation, municipality-years were included in this analysis only if there was at least 1 death at any age recorded to increase the likelihood that deaths were properly recorded. Forced displacement caused significant migration patterns and population changes over the study period. However, deaths recorded were attributed to the municipality of residence of the decedent, regardless of where the death occurred. As with conflict events, some child and infant deaths were undoubtedly not captured by official death records, but these bias our results toward the null, so true associations may be larger than estimated. Finally, these findings may not be generalizable to other conflict-affected settings due to the unique nature and contextual factors of Colombia’s armed conflict.

Although some prior studies have shown disproportionately poor child health outcomes in countries that have experienced conflict, to our knowledge, this cohort study is one of the first studies to quantify the association between armed conflict and child mortality in Colombia.^7,40^ Colombia’s conflict, which has lasted over half a century, is more long term, complex, and localized than many of the conflicts that have been studied in association with child health. One hallmark of Colombia’s conflict is the dynamic spatiotemporal distribution of violence: armed guerrilla groups mostly concentrated in rural areas with geostrategic territorial features, with considerable movement over time.^41,42^ This national ecological cohort study leveraged these changes in conflict intensity over time and space and compared municipalities exposed to conflict with themselves when unexposed to conflict, which improves on prior literature by incorporating a counterfactual. An improved understanding of the association between conflict and child mortality may be helpful to inform programs and public health planning as Colombia moves through its postconflict period with the Revolutionary Armed Forces of Colombia and faces new efforts to achieve peace agreements with several remaining organized armed groups.^29,43,44^

Beyond mortality, millions of children in Colombia have been harmed by the armed conflict through displacement, kidnappings, sexual violence, recruitment into armed groups, and other means.^12^ The indirect associations of conflict with health tend to be long lasting,^23^ so mortality may be the most measurable outcome by official records but likely belies other negative health outcomes.

Unlike natural disasters, armed conflicts are neither spontaneous nor inevitable. More than two-thirds of the world’s children live in countries with active armed conflict.^1^ It is more important than ever for policy makers, advocates, and health professionals to understand the ways armed conflict directly and indirectly harms child health.

## Conclusions

In this ecological cohort study of all municipalities in Colombia from 1998 to 2019, the presence of armed conflict was associated with a 52% increased risk of child (aged <5 years) mortality and a 61% increased risk of infant (aged <1 year) mortality. Over the 22-year study period, 32% of child deaths and 35% of infant deaths in Colombia were associated with armed conflict.

## Notes

Supplement 1.
eMethods.
**eTable.** Associations Between Armed Conflict Exposure and Child and Infant Deaths, 1998-2019, Varying Parameterizations

Supplement 2.
Data Sharing Statement
