# Supplementary material for: Excess Child Mortality Associated With Colombia’s Armed Conflict, 1998-2019
Source: JAMA Netw Open. 2024 Apr 26;7(4):e248510. doi: 10.1001/jamanetworkopen.2024.8510 (PMC11053377; doi:10.1001/jamanetworkopen.2024.8510)
Supplement: Supplement 1. — eMethods. eTable. Associations Between Armed Conflict Exposure and Child and Infant Deaths, 1998-2019, Varying Parameterizations [file jamanetwopen-e248510-s001.pdf]

Supplementary Online Content

Moe CA, Villaveces A, Montoya P, Rowhani-Rahbar A. Excess child mortality associated with Colombia's armed conflict, 1998-2019. *JAMA Netw Open.* 2024;7(4):e248510. doi:10.1001/jamanetworkopen.2024.8510

eMethods.

eTable. Associations Between Armed Conflict Exposure and Child and Infant Deaths, 1998-2019, Varying Parameterizations

This supplementary material has been provided by the authors to give readers additional information about their work.

© 2024 Moe CA et al. *JAMA Network Open.*

eMethods.

The regression model was constructed such that the parameter μ is the mean incidence rate of child death (i.e., the count of child deaths per number of births (n_i) as the offset):

$$\mu_i = n_i \mu \quad (1)$$

$$\mu_i = \exp\left(\ln(n_i) + \beta_0 + \beta_1 x_{1i} + \beta_2 x_{2i} + \beta_3 x_{3i} + \beta_4 x_{4i} + \beta_5 y_i\right) \quad (2)$$

Where x_{1i} represents the indicator for exposure to armed conflict, and x_{2i}, x_{3i}, and x_{4i} are binary indicators for whether there were hurricanes or flooding, earthquake or volcanic eruption, or droughts, respectively. Fixed effects for year were parameterized categorically as y_i, and standard errors were clustered by department.

The 13 types of events in the RUV are: Abandonment or forced loss of land, combats or terrorist attacks, threats, sexual violence crimes, forced disappearance of family members, forced displacement, homicide/massacres of family members, landmine or improvised explosive device (IED) injuries, kidnapping, torture, child and adolescent recruitment, and other physical or psychological injuries linked to conflict.

© 2024 Moe CA et al. *JAMA Network Open.*

eTable. Associations Between Armed Conflict Exposure and Child and Infant Deaths, 1998-2019, Varying Parameterizations

	**RR**	**(95% CI)**	**RD[†]**	**(95% CI)**
≥ 1 *Conflict Event*				
Child (<5) mortality	1.52	(1.34, 1.72)	3.7	(2.7, 4.7)
Infant (<1) mortality	1.61	(1.43, 1.82)	3.0	(2.3, 3.6)
≥ 10 *Conflict Events*				
Child (<5) mortality	1.51	(1.31, 1.73)	4.3	(2.8, 5.7)
Infant (<1) mortality	1.60	(1.38, 1.85)	3.5	(2.4, 4.6)
*Excluding Large Cities**				
Child (<5) mortality	1.50	(1.33, 1.70)	3.6	(2.6, 4.6)
Infant (<1) mortality	1.59	(1.41, 1.79)	2.8	(2.2, 3.5)

† **Computed per 1,000 births**. RR = relative risk; RD = risk difference; CI = confidence interval
All models adjusted for natural disaster indicators of hurricane/flood, volcano/earthquake, or drought, and year fixed effects.
*Excluding 4 largest cities for all years in the study period 1998-2019: Bogotá, Medellín, Cali, and Barranquilla

© 2024 Moe CA et al. *JAMA Network Open.*
