# Supplementary material for: Excess Child Mortality Associated With Colombia’s Armed Conflict, 1998-2019
Source: JAMA Netw Open. 2024 Apr 26;7(4):e248510. doi: 10.1001/jamanetworkopen.2024.8510 (PMC11053377; doi:10.1001/jamanetworkopen.2024.8510)
Supplement: Supplement 2. — Data Sharing Statement [file jamanetwopen-e248510-s002.pdf]

Data Sharing Statement

Moe. Excess Child Mortality Associated With Colombia's Armed Conflict, 1998-2019. *JAMA Netw Open*. Published April 26, 2024. doi:10.1001/jamanetworkopen.2024.8510

Data
Data available: all study data are open access online
Data types: Data (not involving human participants)
How to access data: caitlin.moe2@ucsf.edu
When available: With publication

Supporting Documents
Document types: None

Additional Information
Who can access the data: Statistical/analytic code available upon request; all data used available online/open access
Types of analyses: NA--open access data
Mechanisms of data availability: NA--open access data
